# Quality Characteristics of Novel Pasta Enriched with Non-Extruded and Extruded Blackcurrant Pomace

**DOI:** 10.3390/molecules27238616

**Published:** 2022-12-06

**Authors:** Dorota Gałkowska, Teresa Witczak, Karolina Pycia

**Affiliations:** 1 Department of Food Analysis and Evaluation of Food Quality, University of Agriculture in Krakow, Balicka 122, 30-149 Krakow, Poland; 2 Department of Engineering and Machinery in Food Industry, University of Agriculture in Krakow, Balicka 122, 30-149 Krakow, Poland; 3 Department of Food Technology and Human Nutrition, Institute of Food Technology, College of Natural Science, University of Rzeszow, Zelwerowicza 4, 35-601 Rzeszow, Poland

**Keywords:** blackcurrant, fiber, pasta, pomace, semolina, texture

## Abstract

Fruit pomace is a valuable by-product in terms of its chemical composition, which potential might be used through transformation of the pomace into food ingredients. The aim of this work was to assess the effect of partial (5% and 10%) substitution of powdered non-extruded or extruded blackcurrant pomace for semolina in pasta formula on nutritional and technological properties of the final product. The pasta was assessed for chemical composition, DPPH antiradical activity, color, cooking and textural properties. Presence of the by-products in the pasta resulted in increased total dietary fiber content (from 1.89 ± 0.06 up to 10.03 ± 0.15 g/100 g, dwb), fat content (from 1.29 ± 0.01 up to 2.70 ± 0.05 g/100 g, dwb) and DPPH antiradical activity (from 253 ± 15 up to 1037 ± 7 µmol TE/g, dwb), as well as in significantly different color (*p* < 0.05) as compared to the semolina-only pasta. The optimal cooking time was shortened by 1.0–1.5 min and by 2.0 min in the case of the lower and higher, respectively, level of pasta supplementation. The water absorption decreased by up to 32% in the enriched pasta. In general, the cooking loss remained unchanged. The uncooked product containing the extruded fruit pomace was characterized by significantly higher breaking strength (*p* < 0.05) as compared to the standard pasta. Presence of the pomace also affected texture of the cooked pasta, increasing its firmness and hardness and, when using the non-extruded pomace, the tensile strength. In our research, we have shown that durum wheat pasta enriched with 5 or 10% of powdered blackcurrant pomace or their extrudates constitute a food product of improved nutritional value and of appropriate textural characteristics, while maintaining culinary properties that meet pasta industry requirements.

## 1. Introduction

Pasta is one of the most popular carbohydrate products in the world [1,2]. It is typically made from semolina or common wheat flour and water, and optionally eggs. It comes in both dry and fresh types. The first type is mostly produced commercially via an extrusion process, producing a number of shapes, while the second one is traditionally produced by hand, sometimes with the aid of simple machines. In terms of nutrition, cooked wheat pasta is an important source of complex carbohydrates, i.e., starch (approximately 73%) and, overall, it is a medium low-glycemic index food [3]. This product is low in fat (approximately 1%) and high in protein (approximately 14%) [4]. Nevertheless, wheat pasta, especially that made from the refined flour, contains low micronutrients (i.e., minerals and vitamins) and dietary fiber. Therefore, a trend in the production of pasta enriched with ingredients that increase its nutritional and functional value has been observed for several years. These ingredients are flours from cereals other than wheat, flours from pseudocereals or plants, cereal brans as well as raw materials from animal sources and vegetables [1,3,5]. In the aspect of creating innovative nutritionally enriched pasta, studies on the use of by-products of other food processes products are also undertaken [1,6,7]. This approach is in line with the European Green Deal politics related to the Farm to Fork Strategy aiming, among others, on reducing the environmental impact of the food processing [8]. One of the most popular by-products of fruit processing is fruit pomace. In the case of processing of colored fruits, such as berries, the amount of pomace may constitute up to 30% of the initial weight of the raw material [9]. Numerous studies have shown that berry fruit pomace is a rich source of polyphenols, including proanthocyanins and anthocyanins [9,10]. This is because these compounds are present in the skins and seeds of the berries. Berry fruit pomace is also characterized by high content of fiber (including pectins), protein, lipids as well as simple carbohydrates, minerals, and vitamins [9,11,12,13]. It is also stated in the literature that fruit fiber has better quality from cereal fiber due to higher soluble fiber content [14]. In several previous studies, the possibility of replacing semolina or common wheat flour by fruit pomace in pasta processing have been revealed. In most of the studies, a grape pomace as a source of mainly polyphenolic compounds that increase the antioxidant activity of pasta was used [7,15,16,17,18,19,20], although less popular raw materials, such as apple pomace [21], mango peel powder [22], and coconut residue [23] were also applied as sources of dietary fiber in wheat pasta production. Pomace-fortified pastas might represent food products attractive for consumers, with increased nutritional and pro-health value as well as with good technological and enhanced sensory attributes provided that a precisely estimated level of flour substitution is used [1]. It has been demonstrated that the main reason for the deterioration of culinary and sensory pomace-enriched pasta quality is the high content of fiber, which interferes with the starch-gluten network and alters water penetration into starch granules [1,6,24]. Therefore, the determination of the effect of functional ingredients on the technological properties of pasta should be carried out in parallel with the estimation of nutritional benefits of the final product. To the best of our knowledge, there are no reports in the literature on the use of blackcurrant pomace and their extrudates as fortifying ingredients of wheat pasta.

The use of by-products of the agri-food industry for food processing allows the food treatment to be carried out within the circular economy with minimizing the amount of waste, and thus it closes the loop in industrial ecosystems [6]. In most cases, raw pomace has high water content (up to 50%) and requires immediate processing in order to be stable for storage [9]. The simplest method of removing water is hot-air convection drying; however, this is an energy-consuming process and thus it is not widely used in fruit-vegetable processing plants. There is, therefore, a need for an alternative, more economical drying process for the pomace. An interesting proposal is the extrusion process, which allows for not only to removing water from the raw material but also to create an attractive structure of the finished product. Moreover, it has been proven that the barothermic treatment combined with mechanical shearing of fruit pomace leads to many changes in qualitative and quantitative composition of the raw material, resulting, for instance, in increased antioxidant activity of the finished product as well as better availability of bioactive macro- and micronutrients [25]. Changes in quantity and proportion of dietary fiber as a result of an extrusion process has also been reported [26,27].

Meeting the needs of the modern consumer requiring nutritional and sensory-attractive pasta products and following the idea of the Green Deal, the aim of this work was to assess the effect of partial substitution of powdered non-extruded or extruded blackcurrant pomace for semolina in pasta formula on nutritional, culinary, and textural properties of the final product.

## 2. Results and Discussion

### 2.1. Chemical Composition

The moisture content of the pasta samples ranged from 7.53 to 10.31 g/100 g (Table 1), and thus it was suitable for pasta preservation. The highest moisture content was found in the pasta containing the non-extruded pomace, while the lowest one in the pasta fortified with the extruded pomace. The final moisture content of the pasta samples is due to the initial moisture content of the pasta and its ability to migrate during drying. Dehydration is mainly controlled by water diffusion [28].

Protein content of the blackcurrant pomace, either the non-extruded or extruded, did not differ significantly (*p* = 0.824) from the protein content of the semolina (Table 1). Blackcurrant pomace has been reported to contain both high amount of protein, such as 15.7 g per 100 g (dwb) [11] or 16.9 g per 100 g (dwb) [29], and low, such as 11.1 or 13.3% wet basis [12]. Thus, the botanical variety of the fruits and technology of juice production seem to be essential factors influencing protein content in a pomace. Our results are in accordance with the study by Witczak et al. [30], where lower protein content was found in extruded blackcurrant pomace as compared to non-extruded one. Nevertheless, the influence of extrusion of fruit pomace on their protein content is ambiguous; for example, Witczak et al. [31] reported slightly reduced protein content in extruded black chokeberry pomace as compared to non-extruded one, while Huang and Ma [32] found no significant difference in protein content between non-extruded and extruded orange pomace.

As a result of the substitution of part of the semolina with the extruded blackcurrant pomace, the protein content of the pasta slightly decreased, while in the case of the non-extruded pomace used it did not change significantly (Table 1). It is assumed that the slight reduction in protein content might result from formation of Maillard reaction products. It should be considered, however, that the applied analytical method allows the determination of the protein content on the basis of the amount of nitrogen released from both protein and non-protein compounds. In a view of the above, it may be presumed that the substitution of the semolina flour with the pomace powder resulted in a change in the qualitative composition of nitrogen compounds in the pasta, while maintaining their total quantity at a similar level.

The blackcurrant pomace contained over seven times higher amount of fat than the semolina, wherein the non-extruded pomace showed the highest fat content (Table 1). These results are consistent with these reported by Witczak et al. [31] for chokeberry pomace. According to Reißner et al. [11], pomace fat content largely depends on its seed content; for instance, these authors determined 20.2 g/100 g, dwb, of fat in blackcurrant pomace powder. As stated by Alba et al. [12], analysis of blackcurrant pomace from two distinct regions revealed considerably large difference in fat content (5.9 and 10.8% wet basis) that could have been due to various seed content. Due to our raw materials’ composition fat content in the pasta increased with the amount of the blackcurrant pomace and the effect was stronger in the case of the non-extruded pomace (Table 1).

The ash content in the blackcurrant pomace turned out to be significantly higher (*p* < 0.05) than that determined in the semolina flour (Table 1). Such an outcome is in accordance with the literature data [11,12,30,33]. In a consequence, presence of the fruit raw material in the pasta resulted in slightly increased (*p* < 0.05) ash content. This effect was more pronounced when the non-extruded pomace was used. In the study made by Witczak et al. [30], extruded blackcurrant pomace had a slightly reduced ash content, while Huang and Ma [32] found no significant differences between non-extruded and extruded orange pomace.

The blackcurrant pomace constituted a high-fiber material (Table 1) and this finding agrees with the literature data [13]. Our results of dietary fiber determination correspond also with the data reported by Alba et al. [12], who found predominance of insoluble dietary fiber (approximately 47%, dwb) in blackcurrant pomace. The extruded pomace was characterized by a slightly lower TDF content than the non-extruded one. It was also observed that extrusion of the pomace resulted in a redistribution of IDF into SDF. This finding is in line with that reported by Huang and Ma [32] for orange pomace. It has been stated in the literature [27] that processing parameters affect the quantity and proportion of dietary fiber. In our study, in a consequence of the above-described dietary fiber content of the raw materials the pasta samples enriched with the pomace showed much higher TDF content than the SP (Table 1). For all the samples, the predominance of IDF was found. Substitution of the non-extruded or extruded blackcurrant pomace for the semolina increased SDF content in the final products from approximately 1.4 times in the EP5 to more than 2.5 times in the P10, and increased IDF content from approximately 3.6 times in the EP5 to more than 7.5 times in the P10. It was also found that with increase in the amount of the non-extruded pomace in the pasta, the percentage of SDF in the amount of TDF decreased (from 27.3 to 22.6%), and the percentage of IDF increased (from 72.7 to 77.4%). In contrary, increase of the amount of the semolina substituted with the extruded pomace from 5% to 10% resulted in slightly increased and decreased percentage of SDF and IDF, respectively (25.1% v. 27.6% and 74.9% v. 72.4%), in the TDF content. According to the EFSA [34], a daily recommended dietary fiber intake for adults is 25 g, therefore 100 g of the SP, P5, P10, EP5 and EP10 meet this demand in approximately 8, 24, 40, 20, and 29%, respectively.

All the pasta products differed from each other in available carbohydrate content (Table 1). The higher the share of the non-extruded or extruded blackcurrant pomace in the pasta, the less available carbohydrates it contained. This finding is in accordance with that of Spinelli et al. [35], where the available carbohydrate steadily decreased with the increase of maize bran flour concentration in durum wheat semolina pasta. In our study, a greater reduction in the amount of the carbohydrates in question was found for the pasta with non-extruded pomace: 6% and 12% for the P5 and P10, respectively, vs. 4% and 7% for the EP5 and EP10, respectively. As a consequence of a lower content of non-carbohydrate components and fiber in the EP5 and EP10 as compared to the P5 and P10 (Table 1), the extruded pomace-based pastas had higher content of easily digestible carbohydrates. As might have been expected, results of correlation analysis revealed that available carbohydrate content of the samples studied were significantly negatively correlated with fat content (r = −0.9691, *p* < 0.05) and with total dietary fiber content (r = −0.9979, *p* < 0.05).

### 2.2. DPPH Antiradical Activity

Antioxidant activity of food raw materials depends on both the quantity and composition of the bioactive compounds [27]. Plant sources, including wheat grains and fruits, contain a wide array of phytochemicals, wherein phenolic compounds are predominant [36,37,38]. Regarding durum wheat semolina, derivatives of benzoic acids, p-coumaric acid and ferulic acid have been identified as the main phenolic acids [36,39]. A comprehensive characterization of the profile of phenolic compounds in blackcurrant pomace was carried out by, among others, Kapasakalidis et al. [40]. According to the above-mentioned authors, the main phenol class present in blackcurrant pomace are anthocyanins, including mainly delphinidin-3-glucoside, delphinidin-3-rutinoside, cyanidin-3-glucoside, and cyanidin-3-rutinoside, followed by flavanols and hydroxycinnamic acids. These compounds are powerful antioxidants and exhibit strong radical scavenging activities [40]. It should also be emphasized that nonphenolic components present in blackcurrants, such as ascorbic acid, might contribute to the radical scavenging activity of the isolated extracts [40]. In our study, the fruit pomace exhibited, as expected, much higher antioxidant properties than the semolina (Table 1). As a result of extrusion, the DPPH^•^ scavenging activity of the blackcurrant pomace was reduced by approximately 11%. According to Leonard et al. [27] the extrusion parameters, particularly barrel temperature and screw speed, have significant influence on the anthocyanin level and thus on antioxidant properties of plant by-products. White et al. [41] reported that antioxidant capacity of extruded cranberry pomace determined using the ORAC assay was higher than that of the non-extruded one; however, the extrusion resulted in significant losses of total anthocyanins. Similarly, in the study made by Witczak et al. [31] significant reduction in anthocyanins content of extruded black chokeberry pomace as compared to the non-extruded one was found. In addition, Schmid et al. [42] stated that contents of thermolabile anthocyanins of pure chokeberry pomace decreased with extrusion temperature and with specific mechanical energy. Michalska et al. [43] examined the effect of drying techniques on total polyphenols content and antioxidant capacity of blackcurrant pomace. The authors found that the type of drying method and the drying parameters significantly influenced the total amount of biologically active components, resulting in decrease of approximately 80% of total polyphenols content and decreased ability to scavenge ABTS radical cations.

In our study, the substitution of the semolina flour with the fruit pomace resulted in significantly increased DPPH^•^ scavenging activity (*p* < 0.05) of the produced pastas (Table 1). The antioxidant activity of the P5 and EP5 pasta extracts increased more than twice and almost three times, respectively. Increasing the percentage of pomace in the pasta from 5 to 10% resulted in an over 1.4-fold increase in the antiradical activity (Table 1). Higher antiradical capacity of the pasta containing extruded pomace compared to these with non-extruded pomace may seem contradictory to the characteristics of the fruit raw material in this respect. However, it should be considered that pasta is a processed product, so the parameters of the processing affect the content and composition of the compounds that determine the antiradical activity of the final product. According to Narwal et al. [37], interactions among nutrients and/or antioxidants and/or oxidants occurring during processing may modify the antioxidant activity of foods. In the case of our products, the compounds produced from Maillard reaction, which possess reducing capacity, might increase the antioxidant activity of the heat-treated pasta [37,41]. It cannot be ruled out that differences in the internal structure of the pasta samples also influenced the different extractability of the compounds with antiradical properties.

### 2.3. Color Parameters

**Figure 1:**
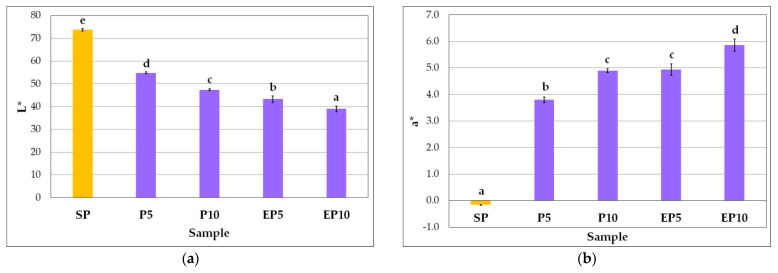

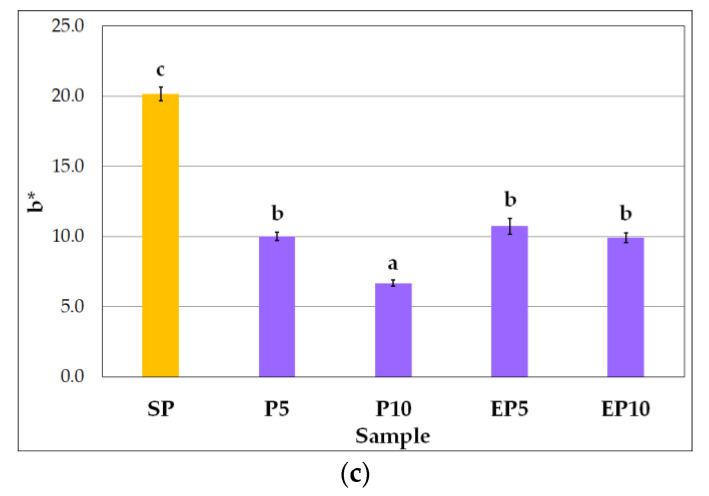
Color parameters of pasta samples: (**a**) L*; (**b**) a*; (**c**) b*. Explanations: SP—semolina-only pasta; P5—pasta with 5% substitution of semolina with non-extruded blackcurrant pomace powder; P10—pasta with 10% substitution of semolina with non-extruded blackcurrant pomace powder; EP5—pasta with 5% substitution of semolina with extruded blackcurrant pomace powder; EP10—pasta with 10% substitution of semolina with extruded blackcurrant pomace powder. Mean values marked with different letters (a–e) are significantly different at significance level of 0.05.

One of the most important attributes of good quality durum wheat pasta is its characteristic yellowness, resulting from high carotenoids content of durum wheat flour [24]. Several studies have demonstrated that modification of pasta formulation due to partial replacement of the base flour with colored fruit pomace resulted in visible color change of the final product [16,19,20,21]. In our study, the semolina-only pasta was characterized by a typical, pale yellow color, while the pomace enriched pasta samples exhibited a rosewood hue. As expected, the SP demonstrated the highest value of coordinate L*, amounting to 73.7 ± 0.63 (Figure 1a), while values of this parameter were significantly reduced (*p* < 0.05) in the pomace enriched pasta samples. The lightness of the enriched pasta samples decreased with the concentration of pomace from 54.8 ± 0.35 to 47.4 ± 0.41 and from 43.3 ± 1.37 to 39.0 ± 1.28 for the P5 and P10 and for the EP5 and EP10, respectively. It was also noted that the extruded pomace pasta samples were darker than their non-extruded counterparts. This observation is consistent with that by Ruskova et al. [44] who found that L* values of extruded apple pomace-wheat semolina blends were lower than L* values of the non-extruded ones. The above-mentioned authors explain this phenomenon by the formation of brown pigments through non-enzymatic reactions occurring during the product processing.

Contrary to the SP sample, the coordinate a* values of all the pomace pasta samples were positive indicating there was a red hue in the color spectrum of these samples (Figure 1b). Increase in the fruit pomace concentration of the pasta enhanced the redness of the pasta, with greater effect observed for the extruded pomace. This observation is in some contradiction to that made by Michalska et al. [43] who found that dehydration of blackcurrant pomace at higher pressure decelerated the release of reddish components from the pomace, resulting in lower a* values as compared to the low-pressure dehydration. Our investigation revealed that the P10 and EP5 pasta samples did not differ significantly in redness. The b* coordinate is a measure of the yellow (positive values) and blue (negative values) colors; therefore, predictably, the SP showed much higher b* value (20.1 ± 0.50) than the other pasta samples (from 6.7 ± 0.23 to 10.8 ± 0.56) (Figure 1c). In the case of the pasta with the non-extruded blackcurrant pomace, the yellowness decreased with concentration of the pomace, while in the case of the EP5 and EP10 the reduction in yellowness was not significant. These observations could be due to the dominance of dark purple pigments in the pasta, thus changes in the b* value have been covered by the changes in the a* value. In this study, significant negative correlation between the parameters a* and b* was found (r = −0.9072, *p* < 0.05). The total color difference (∆*E*) between color of the pasta samples enriched with the non-extruded and extruded pomace at the same level was also calculated. The differences were as follows: 11.6 and 9.1 for the P5-EP5 and the P10-EP10 sample pairs, respectively. It occurred thus that the form of the blackcurrant pomace significantly influenced the color of the resulting pasta, and the effect was even stronger at lower level of the semolina substitution. It can be assumed that darker color and higher redness of the EP5 and EP10 samples as compared to the respective P5 and P10 samples (Figure 1a,b) contributed the most to the above-mentioned differences in color of the samples. Results of the correlation analysis proven that the L* parameter values were significantly negatively correlated with the a* coordinate values (r = −0.9880, *p* < 0.05).

### 2.4. Cooking Properties

In addition to nutritional value, consumer acceptability of pasta is largely influenced by culinary and textural properties, i.e., pasta features that are revealed after hydrothermal treatment of pasta. These properties are regulated mainly by the pasta formula and processing technology. Introduction of an additional enriching ingredient into the basic pasta formulation may result in some interferences in the microstructure of the starch-gluten network, and consequently lead to modification of the culinary and sensory properties (including the textural ones) of the finished product.

One of the basic culinary parameters of pasta is the optimal cooking time (OCT), which is a cooking time required for the starch contained in the pasta to be completely gelatinized. Introduction of the blackcurrant pomace, either the non-extruded or extruded one, to the pasta formula resulted in shortening of the OCT as compared to the OCT of the SP (Table 2). In addition, the OCT was shortened along with the increase in the percentage of the pomace. A number of articles also report a reduction in the optimal cooking time for pomace-fortified pasta [45,46]. The increase in dietary fiber content is indicated as the main cause of this phenomenon. The increase in the soluble and insoluble fiber contents of the pasta (Table 1) was accompanied by a reduction in the OCT (Table 2). It is assumed that fiber interfered with protein-starch matrix development during mixing pasta components and lamination of the dough. The reason for this could lie in a competition of soluble fiber with for water necessary for transformation of protein structure and thus for embedding starch granules in a gluten network [24]. Therefore, after drying, the pasta structure is less uniform and consequently less compact. In a consequence, in our study, the ability of water to penetrate into starch granules during pasta cooking was increased, and thus starch pasting was accelerated [47].

Substitution of the pomace, either extruded or non-extruded, for part of the semolina resulted in a decrease in the quantity of water absorbed by pasta at the OCT and characterized by the WA parameter (Table 2). Water absorbed during pasta cooking is associated with starch swelling and gelatinization [24]. According to Bruneel et al. [48], the restricted water absorption can result from protein polymerization into a protein network that occurs both during pasta drying and the subsequent cooking step. As a consequence, starch granules are entrapped and hindered from swelling during pasta cooking. The gluten dilution effect as well as competition between the fiber and starch for absorption of water should not be ruled out either [49,50]. Negative correlations were found between the WA and the total, soluble, and insoluble dietary fiber content (r = −0.9325, *p* = 0.021; r = −0.9146, *p* = 0.030; r = −0.9294, *p* = 0.022, for TDF, SDF, and IDF, respectively). On the other hand, the available carbohydrate content (Table 1) positively correlated with the WA (r = 0.9354, *p* = 0.020). Assuming that the main component of the available carbohydrates is starch, the above relationship can be interpreted in the context of starch content of the pasta. It should be also noted that the WA values of the pastas containing the extruded pomace (EP5, EP10) were significantly higher than WA values determined in the pastas with the non-extruded pomace (P5, P10). This phenomenon appears to be due to different structures of fiber contained in the non-extruded and extruded blackcurrant pomace, being a consequence of the extrusion process. Huang and Ma [32] reported that extrusion process influenced structural characteristics and chemical composition of fiber contained in an orange pomace, resulting in increased amount of SDF, which retained water within the DF matrix. Similarly, Witczak et al. [30] documented that water absorption index of sour cherry and blackcurrant ground pomace increased as a result of extrusion; however, this parameter determined for the extruded not ground pomace decreased as compared to the respective non-extruded materials.

The swelling index (SI), i.e., the amount of water, in grams, absorbed by one gram of dry pasta, was also determined in the present study (Table 2). In line with the trend observed for the WA, substitution of the non-extruded blackcurrant pomace for 5% or 10% of the semolina resulted in pasta products of significantly reduced values of the SI as compared to the SP. On the other hand, pasta samples enriched with the extruded pomace (EP5, EP10) did not differ significantly in the SI values from the semolina-only pasta. Moreover, there was no significant change in the SI value when the percentage of the pomace (non-extruded or extruded) substitution increased from 5% to 10%.

In many scientific studies it has been claimed that introducing by-products into pasta increases cooking losses [24,45,46]. The reasons for this phenomenon are believed to be the weakening of the starch-protein interactions and diminishing continuity of the protein matrix [24]. As a result, more gelatinized starch as well as other soluble components are leached from the pasta during cooking. The results of our research indicate, however, that the pasta enriched with blackcurrant pomace at the amount of 5% (P5) was characterized by the lowest CL values (Table 2). In other cases, the presence of pomace (non-extruded or extruded) did not significantly change the CL of the pasta as compared to the SP. These observations can be explained by the presence of fat in the pasta. On the one hand, fat contained in the flour could form amylose-lipid complexes, which were responsible for retention of amylose in the product structure during hydrothermal treatment of the pasta [51,52]. On the other hand, an extra fat in the pasta, derived from the blackcurrant pomace, increased the gluten strength by interacting with proteins during the dough mixing process [53]. As reported by Marinelli et al. [7] the observed phenomenon may also be due to the presence of antioxidant compounds from the pomace. These compounds can form complexes with proteins around starch granules, thus encapsulating the starch granules and restricting from leaching. In our research, a slight color of cooking water was observed while cooking the pasta, which means a limited leaching of bioactive compounds, and thus proves their significant retention in the product microstructure. The CL values ranging from 4.70 to 6.19 g dwb/100 g (Table 2) indicate that the acceptable level of culinary losses was achieved for all the pastas, since according to pasta industry guidelines the CL should amount to a maximum of 8% [6].

### 2.5. Textural Properties

Breaking force is a texture parameter determined instrumentally that allows to estimate a behavior of dry uncooked pasta during handling and distribution. The presence of non-extruded or extruded blackcurrant pomace in the composition of the tested pasta increased the breaking force value compared to the SP (Table 2). This means that the modification of the pasta formula resulted in increased mechanical strength of the pasta. In the case of the pasta containing the extruded pomace, the breaking force increased along with the increase in the proportion of the fruit component in the pasta, by 1.3 and 1.7 times for the 5% and 10% substitution level, respectively. Such a statistically significant relationship was not found in the case of the P5 and P10 pasta. An increase in the breaking force of a pasta as a result of replacing 4–6% of wheat flour with grape peel flour was observed by Ungureanu-Iuga and Mironeasa [20]. According to Ogawa et al. [54] the breaking force depends on pasta structure stiffness regulated by the drying temperature. Interestingly, in the present study, a positive correlation was found between the breaking force and the DPPH antiradical activity (r = 0.9736, *p* = 0.005). The dependence of the breaking force of a pasta in the presence of polyphenols (showing antioxidant properties) was also reported by Ungureanu-Iuga and Mironeasa [20]. Antioxidant compounds are believed to contribute to the formation stronger gluten network while pasta dough making and pasta drying [7,20].

Analyzing the results of cutting test of the cooked pasta it was found that the pasta enriched with blackcurrant pomace, either non-extruded or extruded, was characterized by greater both firmness and work of cutting than the SP (Table 2). It can be assumed that mainly the lower amount of absorbed water (WA; Table 2), resulting from structural changes in starch-gluten composite network [55], accounted for the firmer texture of the enriched pasta samples as compared to the SP. The samples containing more pomace showed higher values of the firmness and work of cutting. At the same level of the semolina substitution, higher values of the above-mentioned parameters were characteristic for pasta containing the non-extruded pomace. This relationship is opposite to that found for the breaking strength of the uncooked pasta. It can therefore be concluded that the structural changes caused by the absorption of water by the pasta during cooking dominated the effect of the type of fruit component on the firmness of the pasta. The changes in firmness of the pasta caused by the presence of fruit pomace in their composition are desirable in terms of cooking the pasta products in the al dente way. The results of the correlation analysis showed that both firmness and work of cutting were positive correlated with the TDF content of the pasta (r = 0.9758, *p* = 0.005 and r = 0.9404, *p* = 0.017, respectively). Therefore, our results are inconsistent with the negative effect of plant by-products on pasta texture, including reduction in firmness, documented by many researchers [1,56]. The reason for a deterioration of pasta texture is believed to be mainly modification of the structure of protein network by the particles of a dietary fiber. On the other hand, Ajila et al. [22] and Simonato et al. [46] reported increased firmness for pasta products incorporated with mango peel powder and olive pomace, respectively. According to the latter authors this could be due to the DF particles resistance. It cannot be ruled out that an optimal spatial distribution of the fiber particles in the structure of pasta contributed to the increased firmness of the tested pasta [50,57]. In the present study, the results of the correlation analysis showed that both firmness and work of cutting were positive correlated with fat content of the pasta (r = 0.9481, *p* = 0.014 and r = 0.9341, *p* = 0.02; respectively). This finding allows us to state that the fat also modified the starch-protein structure of the pasta, presumably through the formation of amylose-lipid complexes. It is well established that lipids decrease swelling and solubility of starch [58], contributing to increase the resistance of the pasta to overcook.

The pasta tensile strength determined in the present study may correspond to the elasticity, which is a measure of the degree of extension of the pasta before breaking [45]. The pasta samples containing the non-extruded pomace were distinguished by significantly higher values (*p* < 0.05) of the determined parameter than the other samples; the tensile strength increased by 2 and 1.6 times for the P5 and P10, respectively, as compared to the SP (Table 2). Interestingly, the EP5 was less elastic than the SP, while the higher level of semolina substitution with the extruded pomace did not significantly change the elasticity of the cooked pasta as compared to the SP. Decrease in pasta elasticity as a result of incorporation of 10 or 15% of olive paste flour was reported by Padalino et al. [45]. These authors explain this phenomenon by inclusion of fiber that promoted the formation of discontinuities or cracks in the pasta structure. Similarly, Tudorică et al. [50] noted that durum semolina pasta containing inulin or guar at concentration up to 12.5% showed significantly reduced elasticity compared to the control sample, while the pasta with pea fiber did not differ in this parameter from the control sample. In our research, the difference in pasta behavior in the tensile test between the samples containing non-extruded pomace and these containing the extruded pomace could also be caused by a different degree of pasta swelling, and consequently a different thickness of the cooked pasta ribbons. The P5 and P10 absorbed less water during cooking than the EP5 and EP10, therefore their internal structure was less loosened, and thus more resistant to stretching (which is also reflected in higher values of firmness and work of cutting; Table 2).

## 3. Materials and Methods

### 3.1. Materials

Re-milled durum wheat semolina (Molino F.Lli CHIAVAZZA S.p.A., Casalgrasso, Italy) was bought in a local supermarket. Blackcurrant pomace was kindly supplied by the Fruit and Vegetable Processing Plant (HORTINO ZPOW Leżajsk Sp. z o.o., Leżajsk, Poland). One part of the pomace was freeze-dried at 20–25 °C and 0.520–1.650 mbar for 70 h (Gamma 1-16, Martin Christ Gefriertrocknungsanlagen GmbH, Osterode am Harz, Germany), while the second one was extruded according to the procedure described by Witczak et al. [30]. The non-extruded freeze-dried pomace was then ground in a laboratory knife grinder (A 10, IKA^®^-Werke GmbH & Co. KG, Staufen im Breisgau, Germany) with a simultaneous cooling in order to prevent adverse changes to the product components. The extruded pomace was ground in a laboratory grinder (A 10, IKA^®^-Werke GmbH & Co. KG, IKA, Staufen im Breisgau, Germany). The resulting pomace powders were sieved through 500 µm sieve using a vibratory sieve shaker (Laboratory shaker LPzE-2e, Multiserv-Morek Jan Morek, Brzeźnica, Poland). The materials were stored in tightly closed glass jars at a temperature of 5 °C.

### 3.2. Methods

#### 3.2.1. Pasta Preparation

Five pasta formulations were prepared as follows. The semolina or a mixture of the semolina and fruit pomace was mixed in a planetary mixer (Zelmer ZFP1100B, BSH Sprzęt Gospodarstwa Domowego Sp. z o.o., Warsaw, Poland) with an appropriate amount of water heated to 40 °C. The resulting dough was kneaded for several minutes to a homogeneous consistency. In the semolina dough, only semolina consisted the solid ingredient, while in the fortified pasta, 5% or 10% by weight of the amount of semolina was substituted with the blackcurrant pomace powder (produced from the non-extruded or extruded pomace). The quantitative proportions of the solid ingredient or ingredients to water were selected so that the moisture of the obtained dough was 40%. The pasta dough was then passed through a lab-scale pasta machine (Multipast, Marcato S.r.l., Rome, Italy). Pasta ribbons of approximately. 1 mm thick, 5 mm width and approximately 200-mm length were formed. The pasta ribbons were dried in a laboratory incubator (Climacell 222, BMT Medical Technology s.r.o., Brno, Czech Republic) using the drying procedure described in our previous work [59]). The pasta samples were denoted as follows: SP—semolina-only pasta; P5 and P10—pasta with 5% and 10%, respectively, substitution of semolina with non-extruded blackcurrant pomace powder; EP5 and EP10—pasta with 5% and 10%, respectively, substitution of semolina with extruded blackcurrant pomace powder. Each pasta formulation was prepared in one batch.

#### 3.2.2. Determination of Chemical Composition

Determination of the chemical composition was carried out on the raw materials and pasta samples after grinding the latter in a knife grinder (GM 200; Retsch GmbH, Haan, Germany).

The moisture content was determined by drying at 130 °C for 1.5 h in forced air oven. Protein content was analyzed by the Kjeldahl procedure (using a nitrogen-to-protein conversion factors of 5.7 and 5.3 for semolina and pomace, respectively, considering their mass fraction in the pasta) after sample digestion in concentrated sulphuric acid. Fat content was determined by acid hydrolysis and subsequent Soxhlet extraction with petroleum ether, and ash content after incineration in a muffle furnace for 4 h at 900 °C. Total (TDF), soluble (SDF), and insoluble (IDF) dietary fiber contents were determined by the enzymatic-gravimetric method using a total dietary fiber kit (Megazyme Ltd., Wicklow, Ireland) in accordance with the AOAC 991.43 standard [60]. Protein, fat, ash, and dietary fiber contents of the samples were expressed in g per 100 g, dry weight basis, dwb. Available carbohydrate content, expressed in g per 100 g, dwb, was estimated by difference using the following Formula (1):

Available carbohydrates = 100 − (protein + fat + ash + dietary fiber)(1)

All the determinations were carried out in triplicate.

#### 3.2.3. Determination of Antiradical Activity

The antiradical (antioxidant) activity was determined by the DPPH (2,2-Diphenyl-1-picrylhydrazyl) radical scavenging activity method. Before extraction procedure pasta samples were ground in a knife grinder (GM 200; Retsch GmbH, Haan, Germany) and in a mortal grinder (RM 200, Retsch GmbH, Haan, Germany), and then sieved through 200 µm sieve. The extracts for quantification of the antiradical activity of semolina and pasta were prepared as follows. One gram of the sample was weighted with an accuracy of 0.001 g in a glass vial with a screw cap and then it was mixed with 5 mL of 80% (*v/v*) methanol (Chempur, Poland) and 20 µL of formic acid (Chempur, Piekary Śląskie, Poland). The sample was shaken at room temperature on an orbital shaker (KS 260 basic, IKA, Staufen im Breisgau, Germany) for 45 min at a speed of 350 rpm. The mixture was then centrifuged (MPW-54, MPW Med. Instruments, Warsaw, Poland) for 10 min at 5800 rpm. The supernatant was decanted into a 10 mL glass vial and diluted with 80% (*v/v*) methanol four times, for semolina and semolina-only pasta, or six times, for fortified pasta. The extracts of pomace for the DPPH assay were prepared as follows. Four-tenth of a gram of pomace powder was added with 10 mL of 80% (*v/v*) methanol acidified to pH 2.0 with formic acid, sonicated for 15 min, shaken for 15 min, and then centrifuged for 10 min at 5800 rpm. The supernatant was decanted into a 10 mL glass vial and diluted with 80% (*v/v*) methanol of pH 2.0 four times. A total of 5 mL of 0.1 mM DPPH methanol solution was pipetted into glass vial followed by 300 µL of sample extract or solvent for the blank. The reaction mixture was incubated for 120 min in a darkness at room temperature and then its absorbance at 517 nm was measured against solvent (V-630 UV-Vis spectrophotometer, Jasco Corporation, Kyoto, Japan). The inhibition percentage of the radical scavenging activity (%*I*) was calculated using the Equation (2):

(2) $$\%I=\frac{A_{blank}-A_{sample}}{A_{blank}}\cdot 100$$

where *A_blank_*—absorbance of blank sample; *A_sample_*—absorbance of sample.

A Trolox standard curve in the concentration range of 12–100 µg/mL was utilized for recalculating the %*I* into micrograms of Trolox Equivalent per gram of sample, dwb (µg TE/g, dwb). The measurement was performed in duplicate.

#### 3.2.4. Determination of Color

Color of uncooked pasta ribbons was assessed by means of a Color i5 spectrophotometer (X-rite Incorporated, Grand Rapids, MI, USA) with d/8° sphere geometrics. The CIELab parameters (lightness, L*, and color coordinates, a* and b*) were determined in reflectance measurement mode, at illumination of D65 and an observer angle of 10°. The total color difference (∆*E*) between the pasta samples enriched with the non-extruded pomace (L*_NP_, a*_NP_, b*_NP_) and enriched with the extruded pomace (L*_EP_, a*_EP_, b*_EP_) at the same level was determined according to Equation (3) [61]:

(3) $$\Delta E=\sqrt{{\left(L^{*}{}_{NP}-L^{*}{}_{EP}\right)}^{2}+{\left(a^{*}{}_{NP}-a^{*}{}_{EP}\right)}^{2}+{\left(b^{*}{}_{NP}-b^{*}{}_{EP}\right)}^{2}}$$

#### 3.2.5. Determination of Cooking Properties

Optimal cooking time (OCT) was determined according to the AACC Method 66-50.01 [62]. Procedure for determination water absorption (WA), swelling index (SI), and cooking loss (CL) was performed in duplicate. A 12.5 g of pasta was cooked for the optimal cooking time in 250 mL of boiling tap water, rinsed with cold water, and drained for 5 min. The pasta was than weighted and dried at 105 °C for 2.5 h in an air oven (Venticell 55 Standard, BMT Medical Technology s.r.o., Brno, Czech Republic) and weighted after drying. The pasta cooking water was cooled to room temperature, combined with the pasta rinsing water and made up to a volume of 250 mL with tap water in a measuring cylinder. The liquid was pipetted twice into weighing vessels and then dried in an air oven at 105 °C until constant weight.

The WA (g of water per 100 g of pasta) was calculated using Equation (4):

(4) $$\mathrm{WA}\;\left(g/100g\right)=\frac{\mathrm{weight}\;\mathrm{of}\;\mathrm{cooked}\;\mathrm{pasta}\;\left(g\right)-\mathrm{weight}\;\mathrm{of}\;\mathrm{uncooked}\;\mathrm{pasta}\;\left(g\right)}{\mathrm{weight}\;\mathrm{of}\;\mathrm{uncooked}\;\mathrm{pasta}\;\left(g\right)}\cdot 100$$

The SI (g of water per g of dry weight of pasta) was calculated using Equation (5):

(5) $$\mathrm{SI}\;\left(g/g_{dw}\right)=\frac{\mathrm{weight}\;\mathrm{of}\;\mathrm{cooked}\;\mathrm{pasta}\;\left(g\right)-\mathrm{weight}\;\mathrm{of}\;\mathrm{dried}\;\mathrm{cooked}\;\mathrm{pasta}\;\left(g\right)}{\mathrm{weight}\;\mathrm{of}\;\mathrm{dried}\;\mathrm{cooked}\;\mathrm{pasta}\;\left(g\right)}$$

The CL (g of dry weight of pasta per 100 g of pasta) was calculated using Equation (6):

(6) $$\mathrm{CL}\;\left(g_{dw}/100\;g\right)=\frac{\mathrm{weight}\;\mathrm{of}\;\mathrm{dry}\;\mathrm{residue}\;\left(g\right)}{\mathrm{weight}\;\mathrm{of}\;\mathrm{uncooked}\;\mathrm{pasta}\;\left(g\right)}\cdot \frac{250}{\mathrm{volume}\;\mathrm{of}\;\mathrm{pipetted}\;\mathrm{liquid}\;\left(\mathrm{mL}\right)}\cdot 100$$

#### 3.2.6. Determination of Textural Properties

Textural properties of the pasta were determined using an EZ Test texture analyzer (EZ-LX, Shimadzu, Kyoto, Japan) controlled by the TrapeziumX v. 1.5.2 (Shimadzu, Kyoto, Japan) software.

Three-point bending test of the uncooked pasta ribbon was performed by means of a three-point bending test jig (R2.5 mm × 80 mm). One pasta ribbon of approximately 100 mm length was set on the supporting device with 16 mm span length, and broken by an aluminum upper blade of 80 mm width and 2.5 mm thickness, moving at a speed of 0.5 mm/s. The breaking force (N) expressed as the maximum force required for the fracture was recorded. Testing of the cooked pasta was performed immediately after cooking and draining. The cooked subsamples were kept in a sealed polyethylene box.

Firmness and work of cutting of the cooked pasta were determined according to the AACC Method 66-50.01 [62]. Three pasta samples of 5 mm width, 100 mm length, and 2 mm height were placed in a parallel position onto the measurement plate. An aluminum blade of 80 mm width and 2.5 mm thickness was moved perpendicularly to the plate and the sample was cut. The measurement assay parameters were: pre-test speed 1.0 mm/s, downstroke test speed 0.25 mm/s and downstroke distance 1.2 mm, upstroke test speed 0.5 mm/s and upstroke distance 3.0 mm. The force in function of distance was recorded. The maximum force value was taken as pasta firmness (N), while the area under the positive peak was recorded as work of cutting (mJ).

Tensile strength of cooked pasta ribbon was performed using a noodle tensile jig of a roller type. An approximately 200 mm length pasta ribbon was wrapped onto lower and upper rollers and secured using the tightening force of the pasta itself by moving the upper roller upward with a speed of 1.0 mm/s in a pre-test until tensile force of 0.05 N was achieved. The pasta ribbon was than stretched with a speed of 3.0 mm/s, until it broken. The maximum force value was recorded as a tensile strength (N). All the texture measurements were replicated at least five times.

#### 3.2.7. Statistical Analysis

Statistical analysis of the data was performed using the Statistica 13.1 software package (StatSoft Inc., Tulsa, OK, USA). The data were subjected to a one-way analysis of variance (ANOVA), and the means were compared using Duncan’s test at significance level of 0.05. The Pearson correlation coefficients between the parameters were calculated, and their significance was tested at significance level of 0.05.

## 4. Conclusions

Results of the research allowed for determining the effect of partial substitution of powdered non-extruded or extruded blackcurrant pomace for semolina in pasta formula on nutritional, culinary, and textural properties of the final product. Blackcurrant pomace proved to be a rich source of soluble and insoluble fiber, fat and antioxidants; therefore, durum wheat-blackcurrant pomace pasta was characterized by a significantly higher content of these ingredients as compared to the semolina-only pasta. Using dark colored pomace powders resulted in the supplemented pasta being completely different in color from that of the unenriched pasta. Both a 5% and a 10% proportion of the fruit pomace in the pasta resulted in shortening of the optimal cooking time and reducing the amount of water absorbed by the pasta during cooking, with generally the same level of the cooking loss. The breaking force of the uncooked pasta was higher for the enriched pasta samples, especially for those containing the extruded blackcurrant pomace. The enriched cooked pasta, regardless of the form of blackcurrant pomace and the level of wheat flour substitution, was characterized by greater firmness and greater hardness than the semolina-only pasta. The technological properties of pasta correlated with the content of fat and fiber, the ingredients, which modified the strength of the starch-gluten network in both the uncooked and cooked pasta. In our research, we have shown that durum wheat pasta enriched with 5 or 10% of powdered blackcurrant pomace or their extrudates (based on the amount of the substituted flour) constitute a food product with high nutritious value. Due to the fact that the pasta containing the extruded pomace differed to a lesser extent from the semolina-only pasta in terms of texture characteristics measured after cooking, we suppose that its degree of consumer desirability would be higher than that of the pasta containing the non-extruded by-products.

## Figures and Tables

**Table 1:** Proximate composition of raw materials and pasta samples.

Sample	Moisture Content (g/100 g)	Protein Content (g/100 g, dwb)	Fat Content (g/100 g, dwb)	Ash Content (g/100 g, dwb)	Dietary Fiber Content (g/100 g, dwb)			Available Carbohydrate Content (g/100 g, dwb)	DPPH Antiradical Activity (µg TE/g, dwb)
					TDF	SDF	IDF		
Raw Materials									
Semolina	9.06 ^c^ ± 0.08	14.32 ^a^ ± 0.11	1.61 ^a^ ± 0.02	0.95 ^a^ ± 0.02	1.72 ^a^ ± 0.07	0.54 ^a^ ± 0.06	1.18 ^a^ ± 0.07	81.40 ± 0.13	832 ^a^ ± 13
BcP	3.05 ^b^ ± 0.01	14.41 ^a^ ± 0.26	12.68 ^c^ ± 0.05	3.68 ^b^ ± 0.01	54.53 ^c^ ± 0.13	7.49 ^b^ ± 0.06	47.04 ^c^ ± 0.11	14.70 ± 0.30	10744 ^c^ ± 282
EBcP	2.58 ^a^ ± 0.08	14.47 ^a^ ± 0.41	11.53 ^b^ ± 0.27	3.72 ^b^ ± 0.04	53.22 ^b^ ± 0.13	8.42 ^c^ ± 0.06	44.80 ^b^ ± 0.12	17.06 ± 0.51	9576 ^b^ ± 5
ANOVA–*p*	<0.001	0.824	<0.001	<0.001	<0.001	<0.001	<0.001	-	<0.001
Pasta Samples									
SP	9.93 ^b^ ± 0.00	14.30 ^b^ ± 0.08	1.29 ^a^ ± 0.01	0.87 ^a^ ± 0.05	1.89 ^a^ ± 0.06	0.87 ^a^ ± 0.04	1.03 ^a^ ± 0.03	81.65 ± 0.11	253 ^a^ ± 15
P5	10.31 ^c^ ± 0.02	14.28 ^b^ ± 0.07	1.97 ^b^ ± 0.04	1.05 ^b^ ± 0.05	5.97 ^c^ ± 0.11	1.63 ^c^ ± 0.15	4.34 ^c^ ± 0.04	76.73 ± 0.14	560 ^b^ ± 4
P10	10.20 ^c^ ± 0.14	14.03 ^b^ ± 0.10	2.70 ^d^ ± 0.05	1.19 ^c^ ± 0.01	10.03 ^e^ ± 0.15	2.27 ^e^ ± 0.05	7.77 ^e^ ± 0.11	72.05 ± 0.19	804 ^d^ ± 6
EP5	7.83 ^a^ ± 0.24	13.71 ^a^ ± 0.02	1.91 ^b^ ± 0.07	0.93 ^a^ ± 0.01	4.92 ^b^ ± 0.16	1.23 ^b^± 0.02	3.68 ^b^ ± 0.16	78.53 ± 0.18	706 ^c^ ± 6
EP10	7.53 ^a^ ± 0.12	13.64 ^a^ ± 0.19	2.44 ^c^ ± 0.07	1.07 ^b^ ± 0.02	7.17 ^d^ ± 0.16	1.98 ^d^ ± 0.03	5.19 ^d^ ± 0.13	75.68 ± 0.26	1037 ^e^ ± 7
ANOVA–*p*	<0.001	<0.001	<0.001	<0.001	<0.001	<0.001	<0.001	-	<0.001

Explanations: TDF—total dietary fiber; SDF—soluble dietary fiber; IDF—insoluble dietary fiber; BcP—non-extruded blackcurrant pomace powder; EBcP—extruded blackcurrant pomace powder; SP—semolina-only pasta; P5—pasta with 5% substitution of semolina with non-extruded blackcurrant pomace powder; P10—pasta with 10% substitution of semolina with non-extruded blackcurrant pomace powder; EP5—pasta with 5% substitution of semolina with extruded blackcurrant pomace powder; EP10—pasta with 10% substitution of semolina with extruded blackcurrant pomace powder. Mean values (±standard deviation) in a column followed by different superscript letters (a–e) are significantly different at significance level of 0.05. Statistically significant differences between mean values were determined separately for the raw materials and for the pasta samples.

**Table 2:** Cooking and textural properties of pasta samples.

Sample	Cooking Properties				Textural Properties			
	OCT (min)	WA (g/100 g)	SI (g/g dw)	CL (g dw/100 g)	Breaking Force (N)	Firmness (N)	Work of Cutting (mJ)	Tensile Strength (N)
SP	8.5 ± 0.0	179.3 ^d^ ± 3.0	1.81 ^b^ ± 0.12	5.89 ^b^ ± 0.20	2.76 ^a^ ± 0.30	3.40 ^a^ ± 0.03	1.17 ^a^ ± 0.07	0.23 ^b^ ± 0.01
P5	7.5 ± 0.0	131.3 ^a^ ± 1.9	1.55 ^a^ ± 0.04	4.70 ^a^ ± 0.06	3.39 ^ab^ ± 0.21	4.47 ^c^ ± 0.12	2.04 ^c^ ± 0.10	0.46 ^d^ ± 0.03
P10	6.5 ± 0.0	122.3 ^a^ ± 1.2	1.56 ^a^ ± 0.02	5.86 ^b^ ± 0.21	3.96 ^b^ ± 0.62	5.05 ^d^ ± 0.11	2.24 ^d^ ± 0.01	0.38 ^c^ ± 0.01
EP5	7.0 ± 0.0	151.0 ^c^ ± 3.0	1.90 ^b^ ± 0.03	5.73 ^b^ ± 0.01	3.46 ^b^ ± 0.32	3.87 ^b^ ± 0.19	1.75 ^b^ ± 0.12	0.14 ^a^ ± 0.02
EP10	6.5 ± 0.0	138.0 ^b^ ± 0.3	1.78 ^a b^ ± 0.04	6.19 ^b^ ± 0.13	4.66 ^c^ ± 0.19	4.56 ^c^ ± 0.08	2.05 ^c^ ± 0.13	0.20 ^b^ ± 0.01
ANOVA–*p*	<0.001	<0.001	0.007	<0.001	<0.001	<0.001	<0.001	<0.001

Explanations: OCT—optimum cooking time; WA—water absorption; SI—swelling index; CL—cooking loss; SP—semolina-only pasta; P5—pasta with 5% substitution of semolina with non-extruded black-currant pomace powder; P10—pasta with 10% substitution of semolina with non-extruded blackcurrant pomace powder; EP5—pasta with 5% substitution of semolina with extruded blackcurrant pomace powder; EP10—pasta with 10% substitution of semolina with extruded blackcurrant pomace powder. Mean values (±standard deviation) in a column followed by different superscript letters (a–d) are significantly different at significance level of 0.05.

## Data Availability

The data presented in this study are available on request from the corresponding author.
